# Phytochemical Profiling and Antioxidant Potential of Ultrasonic-Assisted Methanolic Extracts from Different Organs of *Geranium glaberrimum*

**DOI:** 10.3390/biom16101508

**Published:** 2026-10-16

**Authors:** Bedrettin Selvi

**Affiliations:** Department of Biology, Faculty of Arts and Sciences, Tokat Gaziosmanpaşa University, 60250 Tokat, Türkiye; bedrettin.selvi@gop.edu.tr; Tel.: +90-530-692-37-49

**Keywords:** *Geranium glaberrimum*, LC-MS/MS, phenolic compounds, antioxidant activity, ultrasonic extraction, organ comparison

## Abstract

*Geranium glaberrimum* Boiss. & Heldr. is a Turkish endemic species with previously reported phytochemical and biological properties; however, comparative phytochemical and antioxidant data across different plant organs remain scarce. In this study, ultrasonic-assisted methanolic extracts obtained from the flower, leaf, stem, and root were characterized by determining total phenolic content (TPC), total flavonoid content (TFC), antioxidant activities using six complementary assays, relative antioxidant capacity index (RACI), and LC–MS/MS phenolic profiling. A single extract was prepared from each organ, and analytical measurements were performed in technical triplicate. The leaf extract exhibited the highest TFC (53.40 ± 0.15 mg RE/g), whereas the flower extract showed the greatest TPC (252.63 ± 5.73 mg GAE/g). RACI values were highest for the leaf (0.58 ± 0.02) and flower (0.47 ± 0.08), followed by the stem (−0.29 ± 0.19) and root (−0.77 ± 0.04), reflecting their relative positions within the investigated extracts based on the integrated phytochemical parameters and chemical antioxidant assay results. LC–MS/MS profiling targeted 28 phenolic compounds, of which 27 were detected, with hesperidin, hyperoside, gallic acid, quercetin, protocatechuic acid, and kaempferol among the predominant constituents. The antioxidant assays revealed organ-dependent patterns, with flower and leaf extracts generally exhibiting higher radical-scavenging and reducing capacities than the root extract. Exploratory Pearson correlation and hierarchical clustering analyses, based on four organ-level observations, indicated associations between phenolic composition and chemical antioxidant capacity. These findings provide an initial organ-specific phytochemical and antioxidant characterization of *G. glaberrimum* and highlight the need for independent biological replication and further biological validation.

## 1. Introduction

The genus *Geranium* L. (Geraniaceae) comprises approximately 430 species distributed across temperate and mountainous zones worldwide, with a notable concentration of diversity in the Mediterranean basin and southwestern Asia [1]. Members of this genus have a long history of use in folk medicine for their astringent, anti-inflammatory, antimicrobial, and wound-healing properties, attributes widely attributed to their exceptionally rich phenolic chemistry, which encompasses hydrolyzable tannins (geraniin, corilagin, ellagic acid), phenolic acids, flavonols, and flavanonols [2,3]. The pharmacological relevance of *Geranium* spp. has been reinforced by a growing body of recent investigations that document significant antioxidant, cytotoxic, anticholinesterase, and antimicrobial activities in species spanning the geographic range of the genus [4,5,6,7,8,9].

*Geranium glaberrimum* Boiss. & Heldr. is a Turkish endemic member of the genus that grows at altitudes of 1200–2500 m in the Anatolian highlands. It is morphologically distinguished by its glabrous stems, deeply divided leaves, and rose-purple petals, and has been classified within the section *Geranium* alongside other medicinally relevant taxa. The only dedicated phytochemical investigation of this species to date identified gallic acid, ellagic acid, corilagin, geraniin, and quercetin as the major reported phenolic constituents of its ethanolic aerial-part extract, and reported strong antioxidant activity alongside significant tyrosinase-inhibitory effects that were further validated by molecular docking simulations targeting the AbTYR active site [1]. Despite these encouraging findings, organ-specific comparative data—essential for rationalizing ethnobotanical harvesting strategies and guiding extract standardization—are entirely lacking for this species.

The selection of flowers, leaves, stems, and roots was intended to provide a comparative assessment of the principal aboveground and underground organs of *G. glaberrimum*. Because different plant organs may exhibit distinct patterns of secondary metabolite accumulation, their separate investigation allows organ-specific phenolic compositions and chemical antioxidant responses to be characterized without obscuring potential differences through the analysis of combined plant material. This approach also enables the identification of organs that may warrant further phytochemical investigation.

Recent work on phylogenetically and chemically related species underscores the importance of organ-level resolution. Ilic et al. [3] profiled methanolic extracts from aerial and underground parts of eight *Geranium* species collected in southeastern Serbia and demonstrated that underground organs frequently harbour distinct ellagitannin profiles and differential cytotoxicity patterns relative to aerial organs. In the closely related *G. robertianum* L., ultrasound-assisted ethanolic extraction of aerial parts yielded a TPC of 191.7 mg GAE/g alongside substantial DPPH and ABTS radical scavenging capacities, with gallic acid as the overwhelmingly dominant phenolic compound at 43.44 mg/g [7]. Comparative polyphenol analyses of four Romanian wild-grown *Geranium* species (*G. dissectum*, *G. lucidum*, *G. pusillum*, and *G. robertianum*) revealed TPC values ranging from 68.17 to 140.14 mg GAE/g, with gallic and protocatechuic acids consistently aligned with ferric-reducing antioxidant power [5]. Studies on *G. wallichianum* from Pakistan reported TPC of 19.89 mg GAE/g and DPPH half-maximal inhibitory concentration (IC_50_) of 87.94 µg/mL, and demonstrated potent antidiabetic activity attributable to the cumulative phenolic pool [4]. In *G. tuberosum* from Turkey, gallic acid (15,253 mg/kg) dominated the LC-MS/MS profile, and the ethanolic extract showed notable anticholinesterase and antiproliferative activities [6]. Phytochemical comparisons of *G. macrorrhizum* with the related *Pelargonium radula* revealed a richer phenolic diversity in the former, led by rosmarinic acid, *p*-coumaric acid, and catechin-type flavonoids [9]. In the context of fungal endophytes, an endophyte of *G. wallichianum* (*Diaporthe amygdali* GWS39) demonstrated a DPPH IC_50_ of 238.99 µg/mL for its methanolic extract, highlighting the host plant as a reservoir of microbiota with intrinsic bioactive potential [8].

Ultrasonic-assisted extraction (UAE) has emerged as a preferred technique for the recovery of thermolabile polyphenols from plant matrices, offering shorter extraction times, lower solvent consumption, and enhanced mass-transfer efficiency through acoustic cavitation [7]. Methanol was selected as the extraction solvent because of its established suitability for recovering a broad range of polar and moderately polar phenolic compounds from plant matrices for subsequent phytochemical analysis [10,11]. The combination of methanol and ultrasound-assisted extraction was therefore employed to obtain organ-specific extracts under a common extraction protocol, enabling their comparative chemical characterization under consistent experimental conditions. The relative antioxidant capacity index (RACI), which integrates the standardized results of phytochemical parameters (TPC and TFC) and six complementary antioxidant assays into a single composite score, offers a complementary approach for summarizing the relative phytochemical and chemical antioxidant characteristics of the investigated extracts across different analytical methods. Liquid chromatography coupled to tandem mass spectrometry (LC–MS/MS) in multiple reaction monitoring (MRM) mode enables the targeted quantification of multiple phenolic compounds with high analytical selectivity and sensitivity and is widely employed for the quantitative phytochemical characterization of plant extracts [6,7].

The aim of the present study was to comparatively characterize the phenolic composition and chemical antioxidant capacity of ultrasonic-assisted methanolic extracts obtained from the flowers, leaves, stems, and roots of *Geranium glaberrimum*. To achieve this objective, total phenolic and flavonoid contents were determined, 28 target phenolic compounds were investigated by LC–MS/MS, and chemical antioxidant capacities were evaluated using six complementary assays and the Relative Antioxidant Capacity Index (RACI). Additionally, exploratory Pearson correlation analysis and hierarchical clustering were performed to investigate potential associations between phenolic composition and chemical antioxidant assay responses across the four organ-specific extracts.

## 2. Materials and Methods

### 2.1. Plant Material

The plant *G. glaberrimum* Boiss. & Heldr. was collected in full bloom on 1 June 2025, from Otluk Mountain north-facing aspects of calcareous rocks in the Akseki district of Antalya province, Türkiye (37°02′43″ N, 31°54′03″ E) at an altitude of 1450 m. The species was identified by Dr. Bedrettin Selvi, and a voucher specimen with herbarium number (GOPU 8533) has been deposited in the Herbarium of the Faculty of Arts and Sciences at Tokat Gaziosmanpaşa University. In this study, four different parts of the plant were used: flower, leaf, stem, and root. These parts were collected separately during the harvesting process. After collection, the plant material was air-dried in a shaded environment for several weeks. The dried plant parts were then finely powdered using a laboratory mill for further analysis.

### 2.2. Ultrasonic-Assisted Methanolic Extraction

Ultrasonic-assisted methanolic extraction of the flower, leaf, stem, and root materials was performed using an ultrasonic bath. For each organ, 5 g of material was extracted with 100 mL of HPLC-grade methanol (1:20, *w*/*v*) in an ultrasonic bath operating at 40 kHz and 260 W at approximately 30 °C for 60 min [12,13]. Following extraction, the mixtures were filtered through Whatman No. 1 filter paper, and the solvent was removed under reduced pressure using a rotary evaporator with the water bath maintained at 30 °C. The resulting extracts were concentrated and stored at 4 °C until further analyses. The extraction yields were calculated based on the dry weight of the plant material and determined as 4.44%, 7.79%, 3.73%, and 6.62% for flower, leaf, stem, and root, respectively (*w*/*w*). The same extraction conditions were applied to all four plant organs to maintain methodological consistency in the comparative phytochemical and chemical antioxidant analyses.

For each plant organ, a single methanolic extract was prepared from the corresponding dried and powdered plant material. No independent biological or extraction replicates were included in the experimental design. All subsequent phytochemical and antioxidant measurements were performed in technical triplicate using the same organ-specific extract. Accordingly, the three measurements represented analytical repeatability rather than independent biological or extraction replicates.

### 2.3. Determination of the Phenolic Compositions of the Extracts

The total phenolic content of the extracts was determined using the Folin–Ciocalteu assay and expressed as gallic acid equivalents (GAEs), whereas total flavonoid content was measured by the aluminium chloride colorimetric method and reported as rutin equivalents (REs) [14,15,16,17].

The phenolic composition of the extracts was characterized using a validated liquid chromatography–electrospray ionization tandem mass spectrometry (LC–ESI–MS/MS) method, enabling the simultaneous determination of multiple phenolic compounds [18]. The selection of the 28 target analytes was based on an established LC–ESI–MS/MS analytical panel routinely used in our laboratory, the availability of authentic reference standards, and the suitability of the corresponding analyte-specific calibration procedures for reliable quantitative determination. The panel encompasses several major classes of phenolic compounds, including hydroxybenzoic and hydroxycinnamic acids, flavonoid aglycones and glycosides, flavan-3-ols, and selected additional phenolic constituents. Its composition also allows the investigation of phenolic compounds previously reported in Geranium species, including gallic acid, quercetin, and other phenolic acids and flavonoids, while providing broader targeted coverage for comparing the four plant organs [1,3]. Importantly, this predefined panel was intended for targeted quantitative phytochemical characterization rather than comprehensive, untargeted metabolomic profiling. Quantitative analyses were performed on an Agilent Technologies 1260 Infinity liquid chromatography system coupled with a 6420 Triple Quadrupole mass spectrometer.

Chromatographic separation was achieved using a Poroshell 120 EC-C18 column (100 mm × 4.6 mm, 2.7 μm particle size) with a binary mobile phase consisting of solvent A (0.1% formic acid in water) and solvent B (methanol), under gradient elution conditions. The column temperature was maintained at 25 °C, the flow rate was set at 0.4 mL min^−1^, and the injection volume was 2 μL.

Mass spectrometric detection was performed using an electrospray ionization (ESI) source operating in both positive and negative ion modes under multiple reaction monitoring (MRM) conditions. All 28 target phenolic compounds were analyzed using authentic reference standards and analyte-specific calibration curves. Compound identification was based on matching retention times and characteristic multiple reaction monitoring (MRM) transitions with those of the corresponding reference standards, whereas quantification was performed using the respective calibration curves.

Optimized mass spectrometric parameters, including capillary voltage, gas temperature, nebulizer pressure, and compound-specific collision energies, as well as method validation data, are provided in Tables S1 and S2 (Supplementary Material).

### 2.4. Chemical Antioxidant Capacity Assays

The antioxidant capacity of the extracts was evaluated using multiple in vitro assays based on different mechanisms of action. Total antioxidant capacity was determined by the phosphomolybdenum method [19,20]. Radical scavenging activity was assessed using the DPPH assay [21,22] and the ABTS assay [23,24]. Metal chelating activity on ferrous ions was determined according to the method of Dinis et al. [25]. The reducing power of the extracts was evaluated using the CUPRAC method [26] and the FRAP assay [27,28]. All assays were performed with slight modifications of previously reported methods [12,29,30].

Results were expressed as IC_50_ or the concentration required to produce an absorbance of 0.500 (EC_0.5_), as appropriate, and as standard equivalents (e.g., Trolox and EDTA equivalents). Detailed experimental procedures and assay conditions are provided in the Supplementary Materials.

### 2.5. Statistical Analysis

Statistical analyses and descriptive data processing were performed using SPSS version 26.0 (IBM Corp., Armonk, NY, USA). For each plant organ, a single methanolic extract was analyzed, and all phytochemical and antioxidant measurements were performed in technical triplicate (n = 3 technical measurements per extract). Results are presented as mean ± standard deviation (SD), reflecting analytical variability within each extract. Since no independent biological or extraction replicates were available for each organ, inferential comparisons among organs using one-way analysis of variance (ANOVA) and Tukey’s post hoc test were not considered appropriate. Therefore, organ-specific differences were interpreted descriptively rather than as statistically established biological differences.

Pearson correlation analysis was performed using the mean values obtained for the four organ-specific extracts (n = 4 organ-level observations). Technical triplicates were averaged within each organ before correlation analysis to avoid treating non-independent measurements as independent observations. The resulting Pearson correlation coefficients (r) were used for exploratory assessment of associations between phytochemical parameters and antioxidant assay results. Given the small number of organ-level observations and the absence of independent biological replication, these correlations were interpreted descriptively, without claims of statistical significance, predictive capacity, or causality.

Hierarchical clustering of the Pearson correlation matrix was performed using Ward’s linkage method based on Euclidean distance to visualize similarities among the observed correlation patterns. The Relative Antioxidant Capacity Index (RACI) was calculated using an adapted approach based on Sun and Tanumihardjo [31]. Eight parameters were included in the calculation: total phenolic content (TPC), total flavonoid content (TFC), phosphomolybdenum, DPPH, ABTS, CUPRAC, FRAP, and metal-chelating activity. The antioxidant assay results were expressed as Trolox equivalents (TE) or EDTA equivalents (EDTAE), whereas TPC and TFC were expressed as gallic acid equivalents (GAE) and rutin equivalents (RE), respectively. The results of each parameter were standardized using z-scores, and the RACI value for each organ-specific extract was calculated as the arithmetic mean of the eight standardized scores. Since higher values of all eight parameters represented greater phenolic/flavonoid contents or chemical antioxidant capacity, no directional inversion was required. Importantly, IC_50_ and EC_0.5_ values were not included in the RACI calculation. Accordingly, positive and negative RACI values indicate positions above and below the mean of the investigated extracts, respectively, rather than the presence or absence of biological antioxidant activity.

### 2.6. Use of Artificial Intelligence

Artificial intelligence (AI) tools were used solely for language editing and improvement of clarity. AI had no role in the study design, data collection, analysis, interpretation, or conclusions of this work. All content was critically reviewed and approved by the author.

## 3. Results and Discussion

### 3.1. Total Phenolic and Flavonoid Contents

The TPC and TFC values of ultrasonic-assisted methanolic extracts from four organs of *G. glaberrimum* are presented in Table 1.

TPC values varied descriptively among the investigated organs, ranging from 164.04 ± 3.12 mg GAE/g (root) to 252.63 ± 5.73 mg GAE/g (flower). The flower extract exhibited the highest observed TPC, followed by the stem (221.20 ± 10.28 mg GAE/g) and leaf (221.03 ± 4.80 mg GAE/g), which showed nearly identical mean values, whereas the root extract exhibited the lowest TPC under the investigated experimental conditions. These values were numerically higher than those reported for *G. robertianum* aerial-part UAE extract (191.7 mg GAE/g) [7] and for the four Romanian *Geranium* species investigated by Bejenaru et al. [5], whose TPC ranged from 68.17 to 140.14 mg GAE/g. They also exceeded the reported value for the root extract of *G. wallichianum* (19.89 mg GAE/g) [4], although differences in extraction procedures, analytical platforms, reference standards, measurement units, and sample characteristics preclude direct conclusions regarding quantitative superiority among species.

The distribution of total flavonoid content (TFC) among the four organ-specific extracts differed from that of total phenolic content (TPC). The highest observed TFC was recorded in the leaf extract (53.40 ± 0.15 mg RE/g), followed by the flower (33.34 ± 0.09 mg RE/g), stem (27.20 ± 0.01 mg RE/g), and root extracts (3.54 ± 0.36 mg RE/g). These numerical differences are presented descriptively because independent biological and extraction replicates were not available; therefore, statistical significance among plant organs cannot be established. The elevated TFC in the leaf is consistent with the high quercetin (3539 µg/g), hyperoside (3065 µg/g), kaempferol (1540 µg/g), and luteolin (415 µg/g) concentrations subsequently quantified in this organ by LC–MS/MS (Table 2), although the individual contributions of these compounds to the colorimetric TFC measurement were not directly determined. The low TFC observed in the root extract (3.54 mg RE/g) was accompanied by comparatively lower concentrations of several quantified flavonoids than those observed in the aerial-organ extracts.

### 3.2. LC-MS/MS Phenolic Profiling

The targeted LC–ESI–MS/MS approach used in the present study enabled the identification and quantification of a predefined panel of 28 phenolic compounds through comparison with authentic reference standards and the use of analyte-specific calibration curves. In a broader analytical context, mass spectrometry-based approaches, including UHPLC–Orbitrap-MS and UPLC–QTOF-MS combined with molecular networking, have also been applied to characterize complex phytochemical compositions in botanical preparations [32,33].

**Figure 1:**
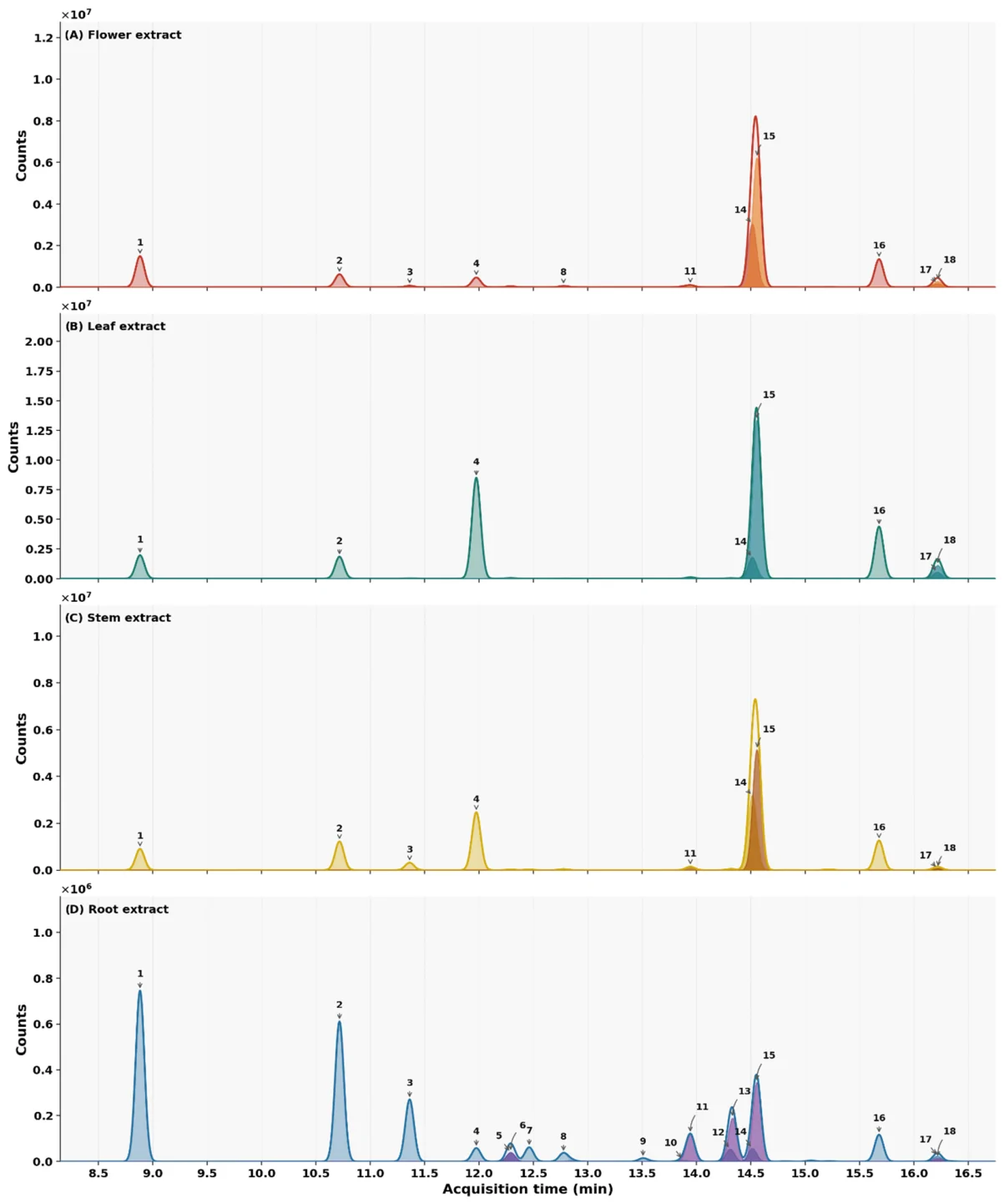
LC–MS/MS chromatograms of ultrasonic-assisted methanolic extracts obtained from different organs of *G. glaberrimum*: (**A**) flower extract, (**B**) leaf extract, (**C**) stem extract, and (**D**) root extract. Peaks were identified according to their retention times and quantified by LC–MS/MS analysis. Only the 18 comparatively abundant phenolic compounds with clearly distinguishable chromatographic peaks are annotated. The remaining nine detected compounds exhibited low-intensity signals that were not readily visible at the displayed scale but were identified and quantified through targeted MRM analysis. Complete quantitative results are provided in Table 2. Labels are as follows: 1, Gallic Acid; 2, Protocatechuic Acid; 3, (+)-Catechin; 4, Chlorogenic Acid; 5, 3-Hydroxybenzoic Acid; 6, 4-Hydroxybenzoic Acid; 7, (−)-Epicatechin; 8, Caffeic Acid; 9, Vanillin; 10, Taxifolin; 11, *p*-Coumaric Acid; 12, Ferulic Acid; 13, Luteolin 7-Glucoside; 14, Hesperidin; 15, Hyperoside; 16, Quercetin; 17, Kaempferol; 18, Luteolin. Different colors are used to distinguish the organ extracts; within each panel, contrasting shades indicate overlapping or closely eluting peaks.

Twenty-seven of the twenty-eight target analytes were detected and quantified in at least one organ extract; 2-hydroxycinnamic acid was not detected (ND) under the experimental conditions in any organ (Table 2). The LC-MS/MS chromatograms for all four organ extracts are presented in Figure 1. Figure 1 displays the chromatographic profiles of the 18 comparatively abundant phenolic compounds whose peaks were clearly distinguishable under the applied chromatographic signal intensity scale. The remaining nine detected compounds were present at substantially lower concentrations, resulting in relatively weak chromatographic signals that were not readily distinguishable in the displayed profiles. Nevertheless, these compounds were identified and quantified using their corresponding MRM transitions, authentic reference standards, and analyte-specific calibration curves. The quantitative results for all 28 targeted analytes, including the compound not detected in any extract, are presented in Table 2.

Compounds were assigned based on retention time matching to authentic reference standards and confirmation through multiple reaction monitoring (MRM) transitions.

The overall phenolic load was dominated by flavonoid glycosides and phenolic acids, with the seven most abundant compounds across all organs—hesperidin, hyperoside, gallic acid, quercetin, protocatechuic acid, chlorogenic acid, and (+)-catechin—collectively accounting for the majority of the quantified phenolic mass. These compound classes are characteristic of the genus *Geranium*, as confirmed across multiple recent comparative studies [3,5,7].

Gallic acid was one of the major phenolic acids detected across the four organ-specific extracts, ranking third among all quantified compounds in the flower (1831 ± 55 µg/g), fourth in the leaf (1797 ± 27 µg/g), sixth in the stem (1445 ± 4 µg/g), and second in the root (516 ± 3 µg/g). In *G. tuberosum*, gallic acid reached 15,253 mg/kg, the highest concentration across all organs of that species [6], a finding broadly consistent with the primacy of gallic acid in Geraniaceae phenolic chemistry. For the four Romanian *Geranium* species, gallic acid in *G. robertianum* was 709.5 µg/g, ranking highest among the four species and correlating directly with FRAP values [5], a relationship also observed in the present study (see Section 3.4). Protocatechuic acid showed an inverse organ distribution relative to gallic acid, with the stem yielding the highest concentration (1898 ± 7 µg/g), followed by leaf (1605 ± 18 µg/g), flower (732 ± 2 µg/g), and root (406 ± 7 µg/g). Chlorogenic acid followed a comparable organ-specific pattern, peaking in the leaf (1927 ± 81 µg/g)—the highest concentration of any phenolic acid in any organ—followed by stem (1013 ± 18 µg/g), flower (145 ± 1 µg/g), and root (11.8 ± 0.3 µg/g); chlorogenic acid was similarly predominant in aerial parts of *G. macrorrhizum* (16.27 mg/g) [3] and *G. macrorrhizum* ethanolic extract (0.96 mg/g DE) [9]. The hydroxycinnamic acids *p*-coumaric acid and ferulic acid were present at moderate concentrations (43.8–123 µg/g and 34.2–55.9 µg/g, respectively), with the stem yielding the highest values for both. Caffeic acid (5.62–48.2 µg/g), rosmarinic acid (2.70–7.44 µg/g), sinapic acid (2.79–6.27 µg/g), and 3,4-dihydroxyphenylacetic acid (2.62–5.59 µg/g) were quantified at lower concentrations across organs. Among the hydroxybenzoic acids, 4-hydroxybenzoic acid, 3-hydroxybenzoic acid, and syringic acid displayed broadly consistent inter-organ distributions, while vanillin was uniquely enriched in the root (29.8 ± 0.2 µg/g).

Turning to the flavonoid fraction, hesperidin and hyperoside emerged as the two dominant compounds overall. Hesperidin was highest in the stem (3150 ± 11 µg/g), followed by flower (2247 ± 17 µg/g), leaf (1028 ± 26 µg/g), and root (18.4 ± 0.2 µg/g). Hyperoside peaked in the leaf (3065 ± 25 µg/g) and stem (2116 ± 5 µg/g), with moderate concentration in the flower (1903 ± 20 µg/g) and negligible content in the root (62.7 ± 1.1 µg/g); hyperoside was also quantified in *G. robertianum* UAE extract at 4.27 mg/g [7], suggesting its potential relevance for comparative phytochemical investigations of *Geranium* species. Quercetin was markedly enriched in the leaf (3539 ± 60 µg/g), and its concentrations in aerial organs of *G. glaberrimum* substantially exceed values reported for *G. robertianum* (1.22 mg/g) [7] and *G. macrorrhizum* (1.23 mg/g) [9]. The previously reported tyrosinase-inhibitory activity associated with quercetin in investigations of G. glaberrimum [1] provides additional context for its potential pharmacological interest; however, the present chemical antioxidant assays do not establish the therapeutic efficacy or safety of the investigated organ-specific extracts. Kaempferol was highest in the leaf (1540 ± 12 µg/g), consistent with *G. tuberosum* (3292.65 mg/kg) [6], while luteolin peaked in the leaf (415 ± 14 µg/g) and luteolin 7-glucoside was notably enriched in the root (21.0 ± 0.5 µg/g)—a reversal of the pattern observed for the aglycone. The flavan-3-ols (+)-catechin and (−)-epicatechin exhibited striking inverse distributions: (+)-catechin was highest in the stem (1833 ± 33 µg/g) and root (658 ± 11 µg/g), but low in the leaf (76.9 ± 0.2 µg/g), while (−)-epicatechin was concentrated in the root (38.6 ± 0.1 µg/g) and stem (36.5 ± 0.1 µg/g), but minimal in aerial organs. The observed differences in (+)-catechin and (−)-epicatechin concentrations among the four organ-specific extracts may reflect variation in phenolic composition; however, the absence of independent biological and extraction replicates precludes definitive conclusions regarding organ-dependent regulation of biosynthetic pathways. Previous investigations have also reported differences in phenolic profiles between aerial and underground organs of several *Geranium* species [3].

The comparatively higher concentrations of several flavonols and hydroxybenzoic acids observed in the aerial-organ extracts may be consistent with the established physiological roles of these compounds in plant responses to ultraviolet (UV) radiation, oxidative stress, and biotic challenges [34,35]. However, the present targeted LC–MS/MS analysis provides compositional information only and does not directly establish organ-specific biosynthetic regulation, metabolite accumulation mechanisms, or physiological functions. Further investigations integrating comprehensive metabolomic profiling with complementary molecular and biochemical approaches would be required to explore these mechanisms. For example, LC–MS-based metabolomic profiling combined with pathway analysis has been employed to investigate metabolic alterations in other biological systems [36], illustrating the additional analytical approaches that may be required to move beyond compositional characterization toward mechanistic interpretation.

Additional phenolic compounds quantified at comparatively lower concentrations included taxifolin (17.2 µg/g in flowers), pinoresinol (19.2 µg/g in flowers), apigenin (2.02–4.30 µg/g), apigenin 7-glucoside (1.16–3.39 µg/g), verbascoside (3.73–7.24 µg/g), and eriodictyol (2.31–22.7 µg/g). The detection of verbascoside in all four organ-specific extracts (3.73–7.24 µg/g extract) is noteworthy, as this compound was not reported in the previously published phytochemical investigation of *G. glaberrimum* [1] and may represent an additional contribution to the known phenolic profile of this species.

### 3.3. Antioxidant Activity and Relative Antioxidant Capacity Index

The antioxidant activities of the four organ extracts, expressed as Trolox equivalents (mg TE/g extract) for the phosphomolybdenum, DPPH, ABTS, CUPRAC, and FRAP assays, and as mg EDTA equivalents (EDTAE)/g extract for metal chelating activity, are presented in Table 3. The corresponding IC_50_ and EC_0.5_ values (mg/mL) together with those of the Trolox or EDTA reference standards are presented in Table 4. The six assays collectively probe distinct mechanistic pathways—SET-based reduction (phosphomolybdenum, CUPRAC, FRAP), HAT-based radical quenching (DPPH, ABTS), and transition metal coordination (metal chelating)—thus offering a mechanistically comprehensive antioxidant profile.

All four organ extracts exhibited high phosphomolybdenum-based antioxidant capacity, with the pattern being root (938 ± 16 mg TE/g) > flower (894 ± 13 mg TE/g) ≈ stem (885 ± 10 mg TE/g) > leaf (875 ± 8 mg TE/g), although the numerical differences among extracts were relatively modest and were interpreted descriptively because independent biological and extraction replicates were not available. The root’s relatively strong total antioxidant power (TAP), despite its low TPC and TFC, may be associated with the presence of reducing constituents, including (+)-catechin (658 µg/g), (−)-epicatechin (38.6 µg/g), and vanillin (29.8 µg/g). However, the individual contributions of these compounds to the phosphomolybdenum assay response were not directly evaluated in the present study. EC_0.5_ values for the phosphomolybdenum assay ranged from 0.416 ± 0.007 mg/mL (root) to 0.446 ± 0.004 mg/mL (leaf), all somewhat higher than the Trolox standard (0.385 ± 0.028 mg/mL), indicating that all extracts approached but did not fully match the reference standard’s potency per unit mass.

In the DPPH radical scavenging assay, the leaf extract demonstrated the strongest observed capacity (722 ± 24 mg TE/g, IC_50_ = 0.319 ± 0.011 mg/mL), followed by flower (656 ± 6 mg TE/g, IC_50_ = 0.350 ± 0.003 mg/mL), stem (555 ± 1 mg TE/g, IC_50_ = 0.414 ± 0.001 mg/mL), and root (392 ± 10 mg TE/g, IC_50_ = 0.587 ± 0.016 mg/mL), indicating a descriptive ranking of DPPH radical-scavenging capacity among the four investigated organ-specific extracts. The higher observed DPPH radical-scavenging capacity of the leaf extract may be associated with its high quercetin (3539 µg/g), kaempferol (1540 µg/g), hyperoside (3065 µg/g), and chlorogenic acid (1927 µg/g) contents, although the individual contributions of these compounds were not directly evaluated in the present study. In *G. wallichianum* root extract, a DPPH IC_50_ of 87.94 µg/mL was reported [4]; while unit conversion and matrix differences preclude direct comparison, the rank order and overall magnitude of DPPH activity in *G. glaberrimum* aerial organs is consistent with the high phenolic density documented in this species. *Geranium glaberrimum* aerial-part ethanolic extract showed >89% DPPH inhibition at 2000 µg/mL [1], further corroborating the potent radical scavenging capacity of this species.

Unlike DPPH, the ABTS radical cation scavenging assay showed relatively modest numerical differences among the four investigated organ-specific extracts: flower (738 ± 73 mg TE/g), leaf (746 ± 31 mg TE/g), stem (653 ± 16 mg TE/g), and root (611 ± 20 mg TE/g). IC_50_ values followed the same pattern (0.216–0.264 mg/mL), and the relatively high standard deviation observed for the flower extract reflects variability among technical measurements rather than biological variability. The comparatively narrow range of ABTS responses, relative to the more pronounced differences observed in DPPH, FRAP, and CUPRAC assays, may suggest that ABTS-reactive constituents are distributed across the investigated organ extracts, including the root. However, these differences are interpreted descriptively because independent biological and extraction replicates were not available. For comparison, Feher et al. [7] reported an ABTS radical-scavenging capacity of 3.159 mmol TE/g extract (equivalent to 790.67 mg TE/g extract) for an ultrasound-assisted ethanolic extract of G. robertianum. Although this finding provides additional evidence of chemical radical-scavenging capacity in another Geranium species, differences in plant species, extraction conditions, and analytical procedures limit direct quantitative comparisons with the present results.

For the CUPRAC assay, values decreased monotonically from flower (692 ± 21 mg TE/g, EC_0.5_ = 0.242 ± 0.007 mg/mL) to leaf (652 ± 9 mg TE/g), stem (622 ± 26 mg TE/g), and root (609 ± 11 mg TE/g, EC_0.5_ = 0.275 ± 0.005 mg/mL). The flower’s lead in CUPRAC, despite its lower TFC relative to the leaf, may reflect the additive contribution of its high gallic acid, hesperidin, and protocatechuic acid concentrations to cupric ion reduction. The FRAP assay yielded a clear descriptive ranking among the four investigated organ-specific extracts: flower (755 ± 7 mg TE/g, EC_0.5_ = 0.065 ± 0.001 mg/mL) > leaf (704 ± 17 mg TE/g) > stem (640 ± 5 mg TE/g) > root (436 ± 2 mg TE/g, EC_0.5_ = 0.112 ± 0.001 mg/mL), although these numerical differences were not subjected to inferential statistical comparisons because independent biological and extraction replicates were unavailable. FRAP was strongly correlated with TPC, an association also reported by Bejenaru et al. [5] for their four Romanian *Geranium* species, where a perfect mirroring of FRAP values by TPC was observed (*G. robertianum* > *G. pusillum* > *G. lucidum* > *G. dissectum*). The FRAP EC_0.5_ values of the organ-specific extracts ranged from 0.065 to 0.112 mg/mL, exceeding that of the Trolox standard (0.047 ± 0.002 mg/mL) and therefore indicating lower iron-reducing potency than Trolox under the experimental conditions.

Metal chelating activity, expressed as mg EDTAE/g extract, showed a substantially different inter-organ pattern compared to all radical scavenging assays: leaf (14.53 ± 0.08 mg EDTAE/g) ≈ root (14.32 ± 0.02 mg EDTAE/g) > stem (14.16 ± 0.02 mg EDTAE/g) > flower (14.11 ± 0.07 mg EDTAE/g). However, the observed numerical differences were relatively small and were interpreted descriptively without inferential statistical comparisons. IC_50_ values ranged from 1.003 ± 0.006 mg/mL (leaf) to 1.033 ± 0.005 mg/mL (flower), all dramatically higher than the EDTA reference standard (0.014 ± 0.001 mg/mL), reflecting the fundamentally lower metal chelating efficiency of phenolic-based chelators relative to the polyaminocarboxylic acid standard. The relatively uniform chelating activity across the investigated organ extracts, together with the weak exploratory associations observed with TPC and TFC, may suggest that specific structural features of individual phenolic constituents, rather than total phenolic content alone, influence metal-chelating responses under the experimental conditions.

**Figure 2:**
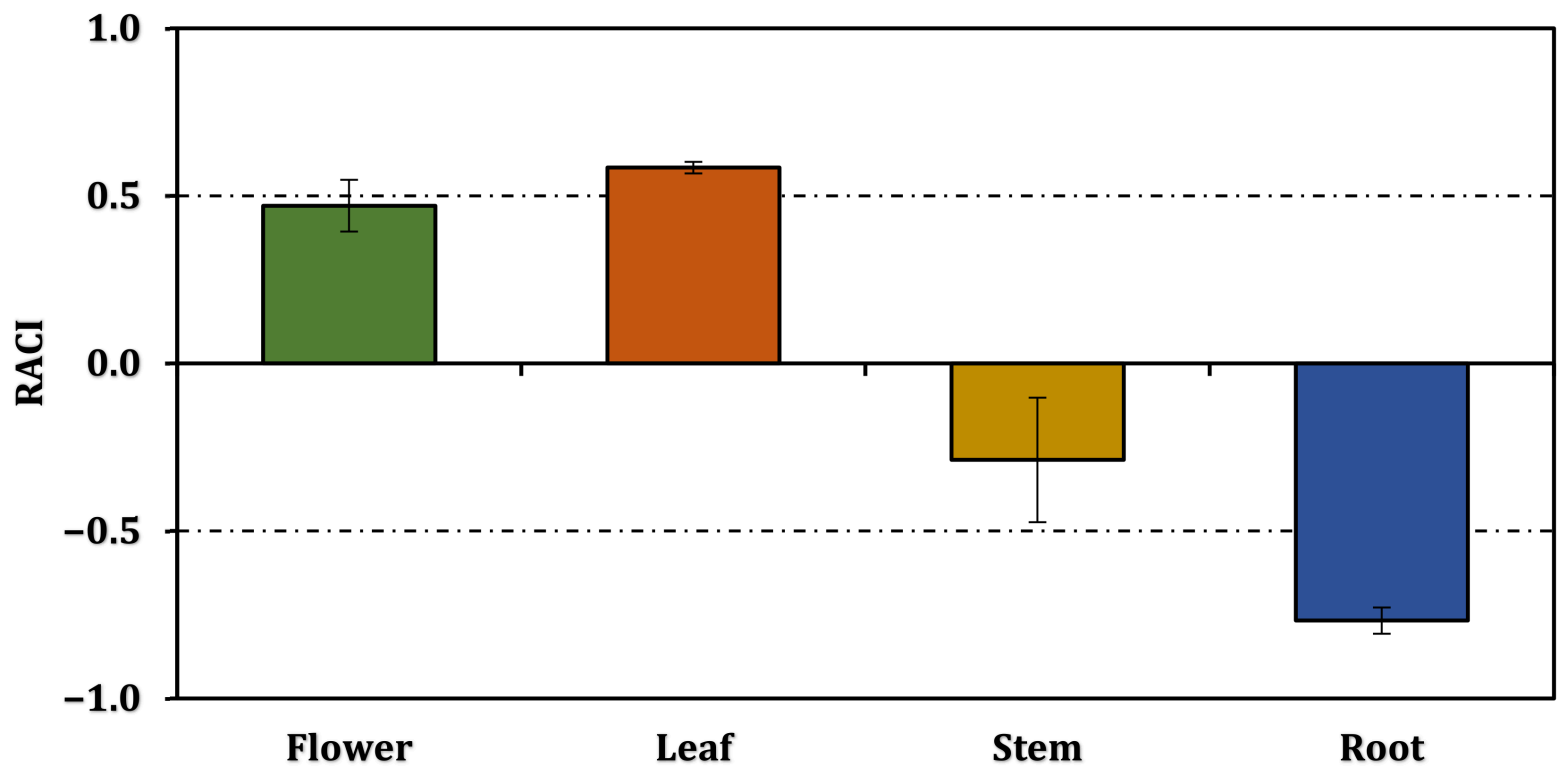
Relative Antioxidant Capacity Index (RACI) values of ultrasonic-assisted methanolic extracts obtained from different organs of *G. glaberrimum*. RACI values were calculated as the arithmetic mean of eight standardized parameter scores, including TPC, TFC, and six antioxidant assays expressed as Trolox or EDTA equivalents. IC_50_ and EC_0.5_ values were excluded from the calculation; therefore, no directional inversion was necessary. RACI calculations were performed separately for three sets of technical measurements, and the results are expressed as mean ± SD of the three resulting RACI scores (n = 3 technical measurements). All measurements were performed on a single methanolic extract prepared from each plant organ. Technical replicates represent analytical repeatability rather than independent biological or extraction replicates. Accordingly, differences in RACI values among organs are presented descriptively, without inferential statistical comparisons.

RACI values, computed by integrating the standardized results of eight parameters (TPC, TFC, and six complementary antioxidant assays), were positive for the flower (0.47 ± 0.08) and leaf (0.58 ± 0.02)—the two organs with the highest combined phenolic and antioxidant capacity—and negative for the stem (−0.29 ± 0.19) and root (−0.77 ± 0.04), indicating their relative positions within the investigated set of organ-specific extracts rather than statistically established differences in biological antioxidant activity. The leaf exhibited the highest observed RACI value, despite not having the greatest TPC, which may be associated with its comparatively high flavonoid content and radical-scavenging responses. However, the contribution of individual phenolic compounds to the integrated antioxidant index was not directly established. The root’s negative RACI value indicates a lower relative position within the integrated assay results, despite exhibiting the highest phosphomolybdenum-based antioxidant capacity among the investigated extracts (Figure 2). Importantly, a negative RACI value does not indicate an absence of chemical antioxidant activity or establish poor biological antioxidant efficacy; it only reflects a below-average composite score relative to the other extracts included in the present comparison. Because RACI values were derived from technical measurements of a single extract per organ, the observed differences should be interpreted descriptively and cannot be generalized to independent biological samples. The RACI approach provides a complementary means of integrating multiple chemical antioxidant assays into a standardized index for comparing the relative antioxidant capacities of the investigated extracts, although it does not directly establish biological antioxidant efficacy [31].

Although the observed phenolic profiles and chemical antioxidant responses suggest that the investigated extracts may warrant further pharmacological or nutraceutical investigation, these findings do not demonstrate therapeutic efficacy, bioavailability, or safety. The present study did not include cellular or in vivo efficacy assessments, toxicological investigations, or pharmacokinetic evaluations. Importantly, previous reviews of other medicinal plants have highlighted that phytochemical richness and reported biological activities do not necessarily imply safety. For example, drug metabolism- and transport-related mechanisms have been implicated in the hepatotoxicity of *Pleuropterus multiflorus* root [37], while the pharmacological and toxicological properties of *Ruta graveolens* have been critically reviewed, emphasizing the need for comprehensive safety evaluation [38]. These studies provide general examples of the importance of safety assessment and should not be interpreted as evidence of toxicity in *G. glaberrimum*. Therefore, further investigations of the present extracts should include appropriate cytotoxicity, toxicity, bioavailability, and biological efficacy assessments before any potential pharmaceutical or nutraceutical applications can be considered.

### 3.4. Hierarchically Clustered Pearson Correlation Analysis

**Figure 3:**
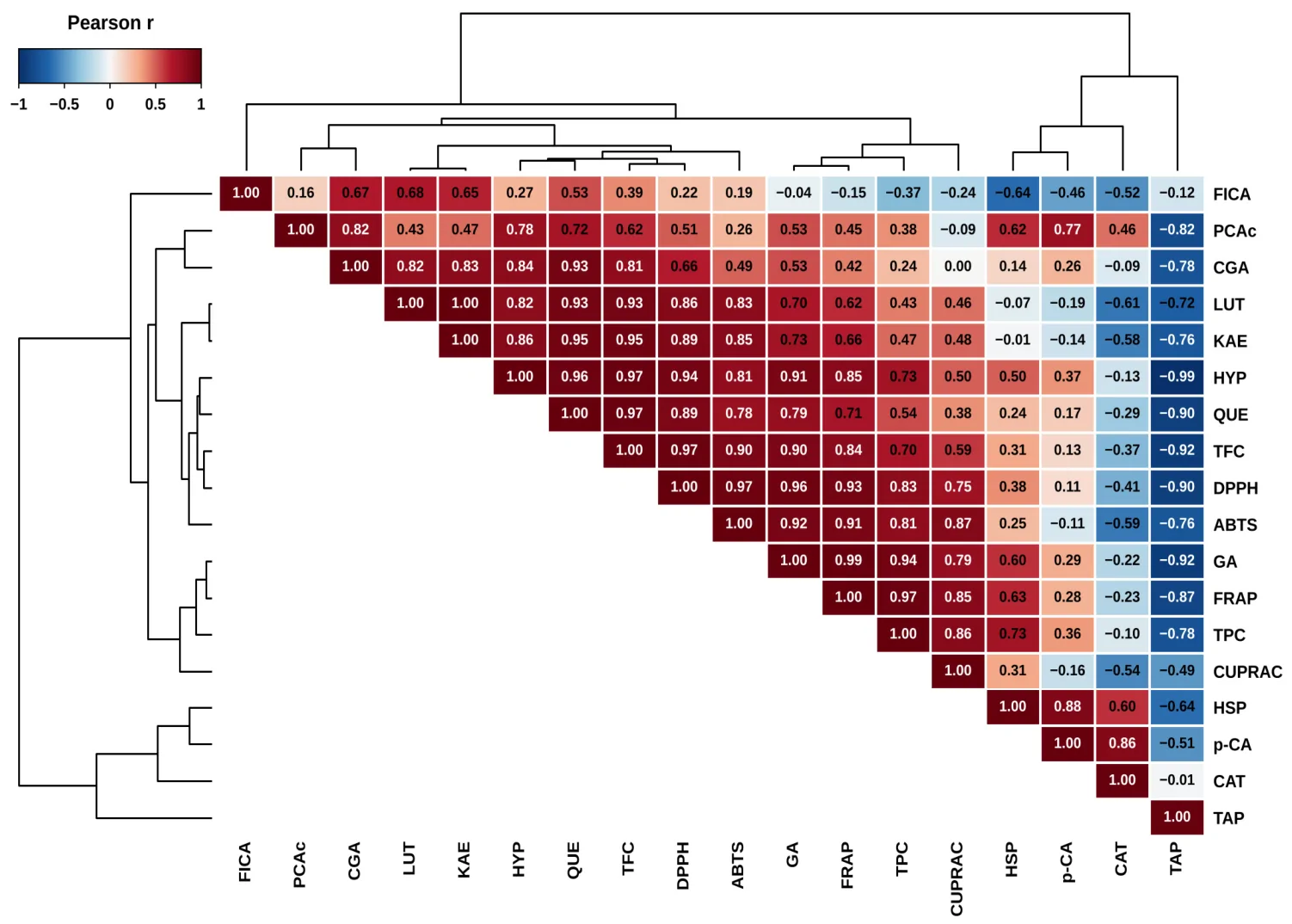
Hierarchically clustered Pearson correlation heatmap showing the relationships among antioxidant assays, total phenolic content (TPC), total flavonoid content (TFC), and major phenolic compounds quantified in *G. glaberrimum* extracts. Pearson correlation coefficients (r) were calculated using four organ-specific mean values (flower, leaf, stem, and root; n = 4), each derived from three technical replicate measurements of a single extract. Pearson correlation coefficients (r) are displayed within each cell and color-coded from negative (blue) to positive (red) correlations. Hierarchical clustering was performed using Ward’s linkage method based on Euclidean distance. Variables with similar correlation profiles were grouped together, as illustrated by the accompanying dendrograms. Because independent biological and extraction replicates were not available, the observed correlations were interpreted as exploratory associations without inferring statistical significance or causality. TAP, phosphomolybdenum assay (total antioxidant capacity); DPPH, 2,2-diphenyl-1-picrylhydrazyl radical scavenging assay; ABTS, 2,2′-azinobis(3-ethylbenzothiazoline-6-sulfonic acid) radical cation scavenging assay; CUPRAC, cupric ion reducing antioxidant capacity; FRAP, ferric reducing antioxidant power; FICA, ferrous ion chelating activity; TPC, total phenolic content; TFC, total flavonoid content; HSP, hesperidin; HYP, hyperoside; GA, gallic acid; QUE, quercetin; PCAc, protocatechuic acid; KAE, kaempferol; CAT, (+)-catechin; LUT, luteolin; CGA, chlorogenic acid; p-CA, *p*-coumaric acid.

The hierarchically clustered Pearson correlation heatmap (Figure 3) revealed distinct groups of antioxidant assays and phenolic compounds sharing similar correlation profiles while simultaneously illustrating the strength and direction of pairwise associations. Within the four investigated organ-specific extracts, positive correlations were observed between TPC and DPPH, FRAP, and CUPRAC activities, suggesting potential associations between total phenolic content and chemical antioxidant capacity rather than establishing direct causal relationships. Conversely, TFC showed a strong positive association with DPPH activity in the exploratory correlation analysis, consistent with the reported antioxidant properties of flavonols such as quercetin and kaempferol identified among the major phenolic constituents of the investigated extracts [3,7]. These correlations were calculated using four organ-level mean values (n = 4) and should therefore be interpreted as descriptive associations rather than statistically confirmed relationships.

It is important to distinguish the concentrations of individual phenolic compounds determined by LC–MS/MS from their correlations with chemical antioxidant assay responses. A compound detected at a relatively high concentration does not necessarily exhibit a strong correlation with antioxidant capacity, and conversely, a strong correlation does not establish a direct functional contribution to the measured activity. Therefore, compound abundance and correlation coefficients were interpreted as separate analytical outcomes. Given that the correlations were calculated from only four organ-level observations (n = 4), the observed associations remain exploratory and should not be considered evidence of causality.

Hierarchical clustering revealed similar correlation patterns among DPPH, FRAP, TFC, gallic acid, hyperoside, quercetin, kaempferol, and luteolin, which were grouped into a closely associated cluster. This clustering pattern suggests potential associations between flavonoid-rich phenolic profiles and radical-scavenging and reducing antioxidant capacities within the four investigated organ-specific extracts, rather than demonstrating direct functional contributions of individual compounds. In contrast, TAP and FICA were positioned in distinct branches of the dendrogram, indicating differences in their observed correlation patterns with the investigated phytochemical parameters. However, these exploratory clustering results should not be interpreted as definitive evidence of distinct underlying antioxidant mechanisms.

Among individual phenolic compounds, gallic acid showed positive correlations with FRAP and DPPH activities across the four investigated organ-specific extracts, a pattern consistent with the association between gallic acid content and FRAP responses reported in four Romanian *Geranium* species [5]. Quercetin and hyperoside concentrations were positively associated with DPPH and ABTS activities, whereas protocatechuic acid showed a positive correlation with FRAP activity. These observed associations may be partly related to structural characteristics of phenolic compounds, including hydroxyl-group number and position, which can influence electron-transfer and hydrogen-donation reactions. Gallic acid contains three phenolic hydroxyl groups, whereas protocatechuic acid, caffeic acid, and the quercetin moiety of hyperoside contain catechol structures that may contribute to their chemical antioxidant properties [5]. The close proximity of these variables within the hierarchical dendrogram indicates similarities in their observed correlation patterns rather than demonstrating coordinated or causal contributions to antioxidant activity. Given the limited number of organ-level observations (n = 4), these relationships should be considered exploratory and hypothesis-generating.

Metal chelating activity showed relatively weak correlations with TPC, TFC, and most individual phenolic compounds across the four investigated organ-specific extracts, suggesting that the observed metal-chelating responses may not follow the same pattern as overall phenolic and flavonoid contents. This observation is consistent with the relatively narrow range of metal-chelating activities recorded among the extracts (Table 3). However, these exploratory correlations do not establish mechanistic independence between metal chelation and other antioxidant processes. The phosphomolybdenum assay (TAP) showed negative correlations with TPC and TFC (r = −0.78 and −0.92, respectively), in contrast to the positive correlations observed for FRAP and DPPH, which may reflect differences in the chemical responses measured by these assays, including potential contributions from reducing constituents not quantified in the targeted phenolic analysis. Nevertheless, these interpretations remain tentative because the correlation analysis was based on only four organ-level observations (n = 4).

The overall correlation pattern observed among the four organ-specific extracts of *G. glaberrimum* showed similarities to previously reported associations between phenolic composition and chemical antioxidant capacity in other *Geranium* species. Higher TPC values tended to be associated with higher FRAP and DPPH responses, while flavonoid content showed positive associations with selected radical-scavenging parameters. In contrast, metal-chelating activity exhibited a distinct correlation pattern, potentially reflecting differences in the chemical properties assessed by the individual antioxidant assays [1,5,7]. Overall, the hierarchically clustered Pearson correlation heatmap provided an integrated visualization of exploratory associations among antioxidant assays and phenolic constituents, highlighting groups of variables with similar correlation patterns rather than demonstrating direct functional contributions or causal relationships. Given the limited number of organ-level observations (n = 4), these findings should be interpreted as preliminary and hypothesis-generating.

It is also important to distinguish the exploratory associations observed in the present chemical assays from potential biological or metabolic effects. Although phytochemical profiling and correlation analysis may help identify associations worthy of further investigation, they cannot establish how individual constituents influence biological pathways. For example, Li et al. [39] investigated relationships among microbial composition, phytosphingosine, and metabolic disorders using metabolite profiling together with complementary experimental approaches. While this study addresses a different biological system and does not provide direct evidence for the activities of the phenolic constituents identified in *G. glaberrimum*, it illustrates the importance of integrating chemical and biological evidence before drawing mechanistic conclusions. Accordingly, the present findings should be viewed as a basis for future targeted biological investigations rather than evidence of metabolic or therapeutic effects.

An important limitation of the present study is the absence of independent biological and extraction replicates. Although all analytical measurements were performed in technical triplicate, these measurements represent analytical repeatability rather than biological variability. Consequently, the observed organ-specific differences cannot be generalized to the broader population of *G. glaberrimum*. Furthermore, Pearson correlation analysis was based on only four organ-level observations, each representing the mean of three technical measurements obtained from a single organ-specific extract, limiting the statistical robustness and generalizability of the observed associations. These results should therefore be regarded as preliminary and hypothesis-generating rather than confirmatory. The importance of distinguishing independent experimental units from technical measurements to avoid pseudoreplication has been emphasized in the statistical literature [40,41]. Future studies incorporating independently collected plant samples and replicate extraction procedures are required to validate the observed organ-specific phytochemical patterns and their relationships with antioxidant capacity.

## 4. Conclusions

This study provides an initial organ-specific phytochemical characterization of *G. glaberrimum* using ultrasonic-assisted methanolic extraction. The flower and leaf extracts exhibited comparatively higher chemical antioxidant capacities according to the observed RACI, TPC, TFC, DPPH, CUPRAC, and FRAP results, while the root extract generally showed lower values in several antioxidant assays but retained a distinctive phenolic signature enriched in (+)-catechin, (−)-epicatechin, luteolin 7-glucoside, and vanillin. Twenty-seven phenolic compounds were quantified by LC–MS/MS, with hesperidin, hyperoside, gallic acid, quercetin, protocatechuic acid, and kaempferol as the principal constituents. These findings suggest descriptive differences in phenolic composition among the four investigated organ-specific extracts.

Pearson correlation and hierarchical clustering analyses revealed exploratory associations between phytochemical composition and chemical antioxidant assay results across the four organ-level observations. In particular, TPC, gallic acid, quercetin, and hyperoside exhibited associations with selected radical-scavenging and reducing antioxidant parameters. However, these correlations do not establish predictive relationships or causal contributions of individual compounds to the measured antioxidant activities. Hierarchical clustering illustrated similarities in the observed correlation patterns, whereas phosphomolybdenum and metal-chelating activities followed distinct descriptive patterns.

Collectively, the results suggest that the aerial organs, particularly the leaves and flowers, may warrant further investigation as sources of phenolic-rich extracts with chemical antioxidant potential. Importantly, the present study was based on a single extract per organ, with analytical measurements performed in technical triplicate. Therefore, the observed organ-specific patterns and correlations should be considered preliminary and cannot be generalized without independent biological and extraction replicates. Importantly, the antioxidant capacities reported in this study were determined exclusively using cell-free chemical assays and should not be interpreted as evidence of biological antioxidant efficacy. The potential antioxidant effects of these extracts in cellular or in vivo systems remain to be established through appropriately designed biological investigations. Potential directions for future research may include investigating the anti-inflammatory, antimicrobial, and enzyme-inhibitory activities of organ-specific extracts, including further characterization of the catechin-containing root extract, as well as evaluating their bioavailability, safety, and antioxidant effects using appropriate cell-based and in vivo models.

## Figures and Tables

**Table 1:** Total phenolic content and total flavonoid content of ultrasonic-assisted methanolic extracts obtained from different organs of *G. glaberrimum*.

Plant Organ	Total Phenolic Content (mg GAE/g Extract)	Total Flavonoid Content (mg RE/g Extract)
Flower	252.63 ± 5.73	33.34 ± 0.09
Leaf	221.03 ± 4.80	53.40 ± 0.15
Stem	221.20 ± 10.28	27.20 ± 0.01
Root	164.04 ± 3.12	3.54 ± 0.36

Values are expressed as mean ± standard deviation (SD) of three technical measurements (n = 3) performed on a single methanolic extract prepared from each plant organ. Technical replicates represent analytical repeatability rather than independent biological or extraction replicates. Accordingly, organ-specific differences are presented descriptively without inferential statistical comparisons. Total phenolic content (TPC) was expressed as mg gallic acid equivalents (GAE)/g extract, whereas total flavonoid content (TFC) was expressed as mg rutin equivalents (RE)/g extract.

**Table 2:** Phenolic compound profile of ultrasonic-assisted methanolic extracts obtained from different organs of *G. glaberrimum* determined by LC–MS/MS analysis.

No	Compounds	Flower	Leaf	Stem	Root
1	Hesperidin	2247 ± 17	1028 ± 26	3150 ± 11	18.4 ± 0.2
2	Hyperoside	1903 ± 20	3065 ± 25	2116 ± 5	62.7 ± 1.1
3	Gallic acid	1831 ± 55	1797 ± 27	1445 ± 4	516 ± 3
4	Quercetin	1464 ± 40	3539 ± 60	1721 ± 19	62.1 ± 0.3
5	Protocatechuic acid	732 ± 2	1605 ± 18	1898 ± 7	406 ± 7
6	Kaempferol	637 ± 8	1540 ± 12	350 ± 2	47.9 ± 0.4
7	(+)-Catechin	325 ± 10	76.9 ± 0.2	1833 ± 33	658 ± 11
8	Luteolin	153 ± 1	415 ± 14	66.1 ± 0.2	5.19 ± 0.09
9	Chlorogenic acid	145 ± 1	1927 ± 81	1013 ± 18	11.8 ± 0.3
10	*p*-Coumaric acid	64.1 ± 0.5	59.4 ± 0.5	123 ± 1	43.8 ± 0.7
11	4-Hydroxybenzoic acid	54.8 ± 0.4	41.6 ± 0.2	50.0 ± 0.5	43.2 ± 0.1
12	Ferulic acid	53.0 ± 0.6	34.2 ± 0.3	55.9 ± 1.0	43.7 ± 0.1
13	Caffeic acid	48.2 ± 0.3	5.62 ± 0.01	43.2 ± 0.1	16.0 ± 0.3
14	3-Hydroxybenzoic acid	34.8 ± 0.2	27.2 ± 0.1	24.2 ± 0.1	30.6 ± 0.3
15	Syringic acid	21.5 ± 0.6	3.53 ± 0.24	11.8 ± 0.1	18.9 ± 0.2
16	Pinoresinol	19.2 ± 0.2	8.43 ± 0.04	13.0 ± 0.1	2.05 ± 0.10
17	Taxifolin	17.2 ± 0.3	1.92 ± 0.10	5.06 ± 0.15	3.61 ± 0.04
18	Rosmarinic acid	7.44 ± 0.03	3.28 ± 0.02	5.80 ± 0.01	2.70 ± 0.06
19	Eriodictyol	7.04 ± 0.35	4.28 ± 0.19	22.7 ± 0.2	2.31 ± 0.01
20	Luteolin 7-glucoside	6.82 ± 0.09	5.28 ± 0.13	10.9 ± 0.1	21.0 ± 0.5
21	Vanillin	5.36 ± 0.39	11.8 ± 0.1	8.51 ± 0.09	29.8 ± 0.2
22	Verbascoside	5.34 ± 0.25	3.89 ± 0.01	7.24 ± 0.14	3.73 ± 0.08
23	Sinapic acid	5.20 ± 0.2	3.48 ± 0.05	6.27 ± 0.05	2.79 ± 0.11
24	(−)-Epicatechin	5.06 ± 0.16	2.14 ± 0.09	36.5 ± 0.1	38.6 ± 0.1
25	3,4-Dihydroxyphenylacetic acid	4.17 ± 0.09	3.17 ± 0.11	5.59 ± 0.16	2.62 ± 0.09
26	Apigenin	3.37 ± 0.09	2.46 ± 0.18	4.30 ± 0.39	2.02 ± 0.05
27	Apigenin 7-glucoside	2.97 ± 0.08	2.12 ± 0.02	3.39 ± 0.32	1.16 ± 0.01
28	2-Hydroxycinnamic acid	ND	ND	ND	ND

Values are expressed as mean ± standard deviation (SD) of three technical measurements (n = 3) performed on a single methanolic extract prepared from each plant organ and reported as µg/g extract. Technical measurements were not considered independent biological or extraction replicates, and no inferential statistical comparisons were made among organs. ND indicates compounds that were not detected under the experimental conditions. Phenolic compounds were identified and quantified by LC–MS/MS using authentic reference standards based on retention times and multiple reaction monitoring (MRM) transitions.

**Table 3:** Antioxidant activities of ultrasonic-assisted methanolic extracts obtained from different organs of *G. glaberrimum*.

Assay	Flower	Leaf	Stem	Root
Phosphomolybdenum assay (mg TE/g extract)	894 ± 13	875 ± 8	885 ± 10	938 ± 16
DPPH radical scavenging (mg TE/g extract)	656 ± 6	722 ± 24	555 ± 1	392 ± 10
ABTS radical scavenging (mg TE/g extract)	738 ± 73	746 ± 31	653 ± 16	611 ± 20
CUPRAC reducing (mg TE/g extract)	692 ± 21	652 ± 9	622 ± 26	609 ± 11
FRAP reducing (mg TE/g extract)	755 ± 7	704 ± 17	640 ± 5	436 ± 2
Metal chelating activity (mg EDTAE/g extract)	14.11 ± 0.07	14.53 ± 0.08	14.16 ± 0.02	14.32 ± 0.02

Values are expressed as mean ± standard deviation (SD) of three technical measurements (n = 3) performed on a single methanolic extract prepared from each plant organ. Technical replicates represent analytical repeatability rather than independent biological or extraction replicates; therefore, differences among organs are interpreted descriptively. Trolox equivalents (TE) were used to express the results of the phosphomolybdenum, DPPH, ABTS, CUPRAC, and FRAP assays. EDTA equivalents (EDTAE) were used for the metal-chelating activity assay. All results are expressed on an extract dry-weight basis (g extract).

**Table 4:** IC_50_ and EC_0.5_ values (mg/mL) for antioxidant assays performed on ultrasonic-assisted methanolic extracts obtained from different organs of *G. glaberrimum*.

Assay (IC_50_/EC_0.5_, mg/mL)	Flower	Leaf	Stem	Root	Standard
Phosphomolybdenum assay	0.436 ± 0.006	0.446 ± 0.004	0.441 ± 0.005	0.416 ± 0.007	0.385 ± 0.028
DPPH radical scavenging	0.350 ± 0.003	0.319 ± 0.011	0.414 ± 0.001	0.587 ± 0.016	0.225 ± 0.016
ABTS radical scavenging	0.219 ± 0.022	0.216 ± 0.009	0.247 ± 0.006	0.264 ± 0.008	0.166 ± 0.010
CUPRAC reducing	0.242 ± 0.007	0.257 ± 0.004	0.270 ± 0.011	0.275 ± 0.005	0.169 ± 0.007
FRAP reducing	0.065 ± 0.001	0.070 ± 0.002	0.077 ± 0.001	0.112 ± 0.001	0.047 ± 0.002
Metal chelating activity	1.033 ± 0.005	1.003 ± 0.006	1.029 ± 0.002	1.017 ± 0.001	0.014 ± 0.001

Values are expressed as mean ± standard deviation (SD) of three technical measurements (n = 3) performed on a single methanolic extract prepared from each plant organ. Technical replicates do not represent independent biological or extraction replicates; therefore, differences among organs are presented descriptively without inferential statistical comparisons. IC_50_ values represent the concentrations required to achieve 50% inhibition in radical-scavenging and metal-chelating assays, whereas EC_0.5_ values represent the concentrations required to produce an absorbance of 0.500 in reducing-power and phosphomolybdenum assays. Lower IC_50_ or EC_0.5_ values indicate stronger activity under the corresponding assay conditions. Trolox was used as the reference standard for the phosphomolybdenum, DPPH, ABTS, CUPRAC, and FRAP assays, whereas EDTA was used for the metal-chelating assay.

## Data Availability

The original contributions presented in this study are included in the article/Supplementary Material. Further inquiries can be directed to the corresponding author.

## Supplementary Materials

The following supporting information can be downloaded at https://www.mdpi.com/article/10.3390/biom16101508/s1, Table S1: ESI–MS/MS parameters and analytical characteristics for the analysis of target analytes in MRM negative and positive ionization mode; Table S2: Calibration curves and sensitivity properties of the method.
