# Supplementary material for: Phytochemical Profiling and Antioxidant Potential of Ultrasonic-Assisted Methanolic Extracts from Different Organs of Geranium glaberrimum
Source: Biomolecules. 2026 Oct 16;16(10):1508. doi: 10.3390/biom16101508 (PMC13702408; doi:10.3390/biom16101508)
Supplement: Supplementary file 1 [file biomolecules-16-01508-s001.zip › biomolecules-4610988-supplementary.pdf]

Supplementary Materials

Section S.1: Analytical methods applied for phytochemical composition and antioxidant activities.

Phytochemical analysis

A simple, rapid, reproducible, and sensitive previously developed and validated LC–ESI–MS/MS method was employed for the targeted determination of 28 phenolic compounds in the present study. The analytical panel comprised 28 selected phenolic standards, and 27 compounds were detected in at least one of the investigated organ extracts. An Agilent Technologies 1260 Infinity liquid chromatography system coupled to a 6420 Triple Quadrupole mass spectrometer was used for quantitative analyses. Chromatographic separation was carried out on a Poroshell 120 EC-C18 (100 mm × 4.6 mm I.D., 2.7 µm) column. The mobile phase configuration (0.1% formic acid/methanol) was selected based on the better chromatographic resolution of isomeric compounds. On the other hand, the selected mobile phase configuration also provided higher sensitivity for many of the phenolic compounds. As a result, the mobile phase was made up from solvent A (0.1%, *v*/*v* formic acid solution) and solvent B (methanol). The gradient profile was set as follows: 0.00 min, 2% B eluent; 3.00 min, 2% B eluent; 6.00 min, 25% B eluent; 10.00 min, 50% B eluent; 14.00 min, 95% B eluent; 17.00 min, 95% B eluent; and 17.50 min, 2% B eluent. The column temperature was maintained at 25°C. The flow rate was 0.4 mL min^{-1} and the injection volume was 2.0 µL. The tandem mass spectrometer was interfaced to the LC system via an ESI source. The electrospray source of the MS was operated in negative and positive multiple reaction monitoring (MRM) mode and the interface conditions were as follows: capillary voltage of −3.5 kV, gas temperature of 300°C and gas flow of 11 L min^{-1}. The nebulizer pressure was 40 psi.

In negative and positive multiple reaction monitoring (MRM) mode, the peaks of the analytes were identified by comparing the retention time, together with the monitoring ion pairs in an authentic standard solution.

Table S1. ESI–MS/MS parameters and analytical characteristics for the analysis of target analytes in MRM negative and positive ionization mode.

Target compounds	Rt (min)	Precursor ion	MRM1 (CE, V)	MRM2 (CE, V)
Compounds analyzed by NI mode				
Gallic acid	8.883	168.9 [M − H]−	125.0 (10)	–
Protocatechuic acid	10.717	152.9 [M − H]−	108.9 (12)	–
3,4-Dihydroxyphenylacetic acid	11.265	167.0 [M − H]−	123.0 (2)	–
(+)-Catechin	11.362	289.0 [M − H]−	245.0 (6)	202.9 (12)
3-Hydroxybenzoic acid	12.290	137.0 [M − H]−	93.0 (6)	–
4-Hydroxybenzoic acid	12.292	136.9 [M − H]−	93.1 (14)	–
Caffeic acid	12.777	179.0 [M − H]−	135.0 (12)	–
Syringic acid	12.849	196.9 [M − H]−	181.9 (8)	152.8 (6)
Vanillin	13.508	151.0 [M − H]−	136.0 (10)	–
Verbascoside	13.576	623.0 [M − H]−	461.0 (26)	160.8 (36)
Taxifolin	13.871	303.0 [M − H]−	285.1 (2)	125.0 (14)
Sinapic acid	13.940	222.9 [M − H]−	207.9 (6)	163.8 (6)
p-Coumaric acid	13.944	162.9 [M − H]−	119.0 (12)	–
Ferulic acid	14.310	193.0 [M − H]−	177.8 (8)	134.0 (12)
Luteolin 7-glucoside	14.331	447.1 [M − H]−	285.0 (24)	–
Rosmarinic acid	14.583	359.0 [M − H]−	196.9 (10)	160.9 (10)
2-Hydroxycinnamic acid	15.032	162.9 [M − H]−	119.1 (10)	–
Pinoresinol	15.053	357.0 [M − H]−	151.0 (12)	135.7 (34)
Eriodictyol	15.215	287.0 [M − H]−	151.0 (4)	134.9 (22)
Quercetin	15.679	301.0 [M − H]−	178.6 (10)	151.0 (16)
Kaempferol	16.214	285.0 [M − H]−	242.8 (16)	229.1 (18)
Compounds analyzed by PI mode				
Chlorogenic acid	11.975	355.0 [M + H]+	163.0 (10)	–
(−)-Epicatechin	12.462	291.0 [M + H]+	139.1 (12)	122.9 (36)
Hesperidin	14.513	611.1 [M + H]+	449.2 (4)	303.0 (20)
Hyperoside	14.555	465.1 [M + H]+	303.1 (8)	–
Apigenin 7-glucoside	14.815	433.1 [M + H]+	271.0 (18)	–
Luteolin	16.218	287.0 [M + H]+	153.1 (34)	135.1 (36)
Apigenin	16.337	271.0 [M + H]+	153.0 (34)	119.1 (36)

R_t, retention time; NI, negative ion; PI, positive ion.

Table S2. Calibration curves and sensitivity properties of the method.

Compounds	Linearity and sensitivity characteristics				
	Range (μg/L)	Linear equation	R^2	LOD (μg/L)	LOQ (μg/L)
Gallic acid	5–500	y = 4.82x − 26.48	0.9988	1.46	4.88
Protocatechuic acid	2.5–500	y = 5.65x − 9.99	0.9990	1.17	3.88
3,4-Dihydroxyphenylacetic acid	5–500	y = 5.13x − 12.39	0.9990	1.35	4.51
(+)-Catechin	10–500	y = 1.45x + 1.95	0.9974	3.96	13.20
Chlorogenic acid	1–500	y = 12.14x + 32.34	0.9995	0.55	1.82
3-Hydroxybenzoic acid	5–500	y = 3.69x − 12.29	0.9991	1.86	6.20
4-Hydroxybenzoic acid	5–500	y = 7.62x + 22.79	0.9996	1.72	5.72
(−)-Epicatechin	5–500	y = 9.11x − 9.99	0.9971	1.85	6.18
Caffeic acid	5–500	y = 11.09x + 16.73	0.9997	3.15	10.50
Syringic acid	10–500	y = 0.74x − 1.54	0.9975	3.75	12.50
Vanillin	50–500	y = 2.02x + 135.49	0.9926	15.23	50.77
Verbascoside	2.5–500	y = 8.59x − 28.05	0.9988	0.82	2.75
Taxifolin	5–500	y = 12.32x + 9.98	0.9993	1.82	6.05
Sinapic acid	5–500	y = 2.09x − 6.79	0.9974	2.64	8.78
p-Coumaric acid	5–500	y = 17.51x + 53.73	0.9997	1.93	6.44
Ferulic acid	5–500	y = 3.32x − 4.30	0.9992	1.43	4.76
Luteolin 7-glucoside	1–500	y = 45.25x + 156.48	0.9996	0.45	1.51
Hesperidin	5–500	y = 5.98x + 0.42	0.9993	1.73	5.77
Hyperoside	2.5–500	y = 16.32x − 1.26	0.9998	0.99	3.31
Rosmarinic acid	1–500	y = 9.82x − 17.98	0.9989	0.57	1.89
Apigenin 7-glucoside	1–500	y = 21.33x − 31.69	0.9983	0.41	1.35
2-Hydroxycinnamic acid	1–500	y = 16.72x − 26.94	0.9996	0.61	2.03
Pinoresinol	10–500	y = 0.80x − 2.69	0.9966	3.94	13.12
Eriodictyol	2.5–500	y = 14.24x − 0.50	0.9998	0.80	2.68
Quercetin	5–500	y = 14.68x − 18.25	0.9997	1.23	4.10
Kaempferol	10–500	y = 0.82x − 3.06	0.9959	3.30	10.99
Luteolin	5–500	y = 8.96x + 26.80	0.9992	1.34	4.46
Apigenin	2.5–500	y = 11.29x + 38.05	0.9987	0.96	3.20

LOD and LOQ: limit of detection and limit of quantification, respectively.

Antioxidant activity

For total phenolic content, sample solution (0.25 mL) was mixed with diluted Folin–Ciocalteu reagent (1 mL, 1:9) and shaken vigorously. After 3 min, Na_2CO_3 solution (0.75 mL, 1%) was added and the sample absorbance was read at 760 nm after 2 h incubation at room temperature. Total phenolic content was expressed as equivalents of gallic acid.

For total flavonoid content, sample solution (1 mL) was mixed with the same volume of aluminium trichloride (2%) in methanol. Similarly, a blank was prepared by adding sample solution (1 mL) to methanol (1 mL) without $AlCl_3$. The sample and blank absorbance were read at 415 nm after 10 min incubation at room temperature. Absorbance of the blank was subtracted from that of the sample. Total flavonoid content was expressed as equivalents of rutin.

Total antioxidant activity of the samples was evaluated using the phosphomolybdenum method. Sample solution (0.2 mL) was combined with 2 mL of reagent solution (0.6 M of sulfuric acid, 28 mM of sodium phosphate and 4 mM of ammonium molybdate). The sample absorbance was read at 695 nm after 90 min of incubation at 95°C.

For 1,1-diphenyl-2-picrylhydrazyl (DPPH) radical scavenging activity, sample solution (1 mL) was added to a 4 mL of 0.004% methanol solution of DPPH. Sample absorbance was read at 517 nm after 30 min of incubation at room temperature in the dark.

For ABTS cation radical scavenging activity, briefly, $ABTS^{\cdot+}$ radical cation was produced directly by reacting 7 mM of ABTS solution with 2.45 mM of potassium persulfate and allowing the mixture to stand for 12-16 h in dark at room temperature. Prior to beginning the assay, ABTS solution was diluted with methanol to obtain an absorbance of 0.700 ± 0.02 at 734 nm. Sample solution (1 mL) was added to ABTS solution (2 mL) and mixed. Sample absorbance was read at 734 nm after 7 min incubation at room temperature.

For metal chelating activity on ferrous ions, briefly, sample solution (2 mL) was added to $FeCl_2$ solution (0.05 mL, 2 mM). The reaction was initiated by the addition of 5 mM of ferrozine (0.2 mL). Similarly, a blank was prepared by adding sample solution (2 mL) to $FeCl_2$ solution (0.05 mL, 2 mM) and water (0.2 mL) without ferrozine. Then, the sample and blank absorbance were read at 562 nm after 10 min of incubation at room temperature.

For cupric ion reducing activity (CUPRAC), sample solution (0.5 mL) was added to a premixed reaction mixture containing $CuCl_2$ (1 mL, 10 mM), neocuproine (1 mL, 7.5 mM) and NH_4Ac buffer (1 mL, 1 M, pH 7.0). Similarly, a blank was prepared by adding sample solution (0.5 mL) to a premixed reaction mixture (3 mL) without $CuCl_2$. Then, the sample and blank absorbance were read at 450 nm after 30 min of incubation at room temperature.

For ferric reducing antioxidant power (FRAP), sample solution (0.1 mL) was added to a premixed FRAP reagent (2 mL) containing acetate buffer (0.3 M, pH 3.6), 2,4,6-tris(2-

pyridyl)-s-triazine (TPTZ) (10 mM) in 40 mM of HCl and ferric chloride (20 mM) in a ratio of 10:1:1 (v/v/v). Then, the sample absorbance was read at 593 nm after 30 min of incubation at room temperature.

The sample concentration required to achieve 50% inhibition in radical-scavenging and metal-chelating assays was defined as IC_{50}, whereas $EC_{0.5}$ was defined as the sample concentration required to produce an absorbance of 0.500 in the reducing-power and phosphomolybdenum assays. The chemical antioxidant capacities of the extracts were expressed as mg standard equivalents/g extract and compared with those of the reference standards, including Trolox and ethylenediaminetetraacetic acid disodium salt (EDTA).
